# 

**Published:** 1889-09-07

**Authors:** 


### NOTICE TO CORRESPONDENTS.

All MS., Letters, Books for Review, and other matters intended for the Editor, should be addressed The Editor, The Lodge, Porchester Square, London, W.

### Notice to Advertisers and Subscribers.

All Advertisements, Orders for Copies of The Hospital, and Business Communications should be addressed to The Publisher, *not to the Editor*, The Hospital, 139-140, Salisbury-court, Fleet-street, E.C.

Vol. V., now ready, 4s., post free. Cases for binding, may be had of the Publisher, at 1s. 6d. each.

### To Residents, Secretaries, and Matrons.

The Editor will be glad to have the Regulations and each Report as issued by Asylums, Hospitals, Convalescent and Nursing Institutions, as well as those of other Charities, and an early copy in duplicate of all Appeals and Festival Notices, etc., addressed to *The Editor, The Lodge, Porchester-square, London, W.* Any superintendent, secretary, matron, or other chief official who regularly sends such information may be registered, on application to the Publisher, to receive free of cost a cover or binding The Hospital in half-yearly volumes.

### Amusements and Relaxation.

### Second Quarterly Word Competition Commenced July 6th, ends September 28th.

Three Prizes of 15s., 10s., 5s., will be given for the largest number of words derived from the words set for dissection.

Proper names, abbreviations, foreign words, words of less than four letters, and repetitions are barred; plurals, and past and present participles of verbs, are allowed. Nuttall's *Standard* dictionary only to be used.

N.B.—Word dissections must be sent in WEEKLY, arranged alphabetically, with correct total affixed.

The word for dissection for this, the *Tenth* week of the quarter, being

"B A R M O U T H."

**Results of Eighth Week.**

| Names. | Aug. 29th. | Totals. | Names. | Aug. 29th. | Totals. |
|---|---|---|---|---|---|
| Nurse Duty | 21 | 481 | Leirion | — | 195 |
| N'importe Qui | 19 | 440 | Matron | — | 174 |
| Jumps | — | 30 | F. C. J. | - | 305 |
| Tinie | — | 186 | Percy Verance | — | 18 |
| Patience | 21 | 487 | Gleniffer | — | 175 |
| Amicus | — | 430 | Gypsye | 21 | 422 |
| Broxbourne | — | 398 | Ladyr | — | 127 |
| Speedwell | 20 | 439 | A. B. | — | 170 |
| Sister | 19 | 463 | Essie | 19 | 380 |
| Lightowlers | 21 | 356 | W. G. R. | 19 | 375 |
| Lauriston | 21 | 345 | Cowboy Bill | — | 179 |
| Rattler | — | 28 | M. L. A. | — | 117 |
| Paignton | — | 334 | Little Tuppy | — | 110 |
| Fassifern | — | 141 | Embryo | — | 56 |
| W. R. E. | 20 | 468 | K. Jarman | — | 43 |
| Esperance | 20 | 480 | Reveal | — | 196 |
| M. W. | 20 | 485 | Jenny Wren | 19 | 372 |
| Isabel | 21 | 454 | Condorcet | 20 | 406 |
| Buxton | — | 167 | Electrician | 20 | 245 |
| Nux Vom | — | 263 | Pearl | — | 132 |
| Qu'appelle | 20 | 419 | R. W. | — | 43 |
| Spes | — | 24 | Nurse Amie | — | 89 |
| Secnarf | — | 229 | Sunshine | 19 | 162 |
| Rover | 20 | 226 | Juvenis | — | 22 |

**Notice to Correspondents.**

N.B.—All letters referring to this page which do not arrive at 139 and 140, Salisbury-court, London, E.C., by the *first post on Thursdays*, and are not addressed PRIZE EDITOR, will in future be disqualified and disregarded

Competitors can enter for *all* quarterly competitions, but no competitor may take more than *two quarterly prizes* during the year.

**Conditions.**

I. Every paper sent in must be clearly written, and one side only must be used. All competitors must mark at the head of their papers the number of their competition.

II. Each paper must be signed by the author with his or her real name and address. A *nom de plume* may be added if the writer does not desire to be referred to by us by his real name. In the case of all prize-winners, however, the real name and address will be published.

III. All communications relating to prize competitions should be addressed to "The Prize Editor, The Hospital, 139 and 140, Salisbury-court, London, E.C." Competitors are requested not to include in letters, etc., to the Prize Editor communications on matters of other business. All letters must be fully prepaid.

IV. The Prize Editor of The Hospital is the sole judge of the competitions, and his decision is in all cases final and without appeal.

V. The Prize Editor reserves the right to publish any paper sent in, whether it receives a prize or not. He does not hold himself responsible for any MSS. sent, nor can he undertake to return rejected competitions.

### Scraps and Gleanings.

There are 300 cases of fever in Cairo Hospital.

The death is announced of Dr. W. C. S. Miller, of Edinburgh.

Sir Andrew Clark has gone to Bewdley for a couple of months.

The late medical staff of Belfast Royal Hospital have been re-elected.

Clacton-on-Sea collected £50 on Hospital Saturday. Bravo, Clacton!

Dr. Corfield reports a further spread of enteric fever at the West End.

The body of the late Colonel Tomline was cremated at Woking last week.

Chelmsford is waking up; it actually proposes to start a Hospital Saturday.

Dr. Oliver Wendell Holmes celebrated his 80th birthday at Boston last week.

The Bill for the establishment of a Dublin Hospital Board has been withdrawn.

Dr. Sophia Unger has been appointed the first lady sanitary inspector in New York.

The ambulance station at Homerton Fever Hospital has been destroyed by fire.

The Ven. Archdeacon Lawrance preached the sermon at St. Albans on Hospital Sunday.

There is a fever epidemic in Constantinople owing to the extreme heat of the weather.

Dr. Tunstall has been appointed assistant medical officer to Birmingham Workhouse.

Mrs. T. Meadows has furnished one of the wards of the new Eye Infirmary at Hereford.

The Thakore of Bhownagur has given Rs.200,000 towards the Bombay Hospital for training nurses.

The insanitary condition of Alexandria was denounced at the late congress as a danger to all Europe.

Dr. Morrice has been appointed resident medical officer of the Homerton Asylums' Board Hospital.

The Working Men's Committee of Hull Hospital issue a special appeal for funds to free their institution from debt.

Dr. Barsham has resigned his connection with Luton Cottage Hospital, and Dr. Clarke has been elected in his stead.

Mr. Charles Brown, house surgeon at St. Thomas's, died last week of diphtheria contracted in the course of his duties.

The first International Congress of Physiologists will be opened at Basle on September 10th, and will sit for three days.

Dr. Fred. H. Heath has resigned his connection with Manchester Royal Infirmary, which has existed for over 30 years.

Two cases of cholera are reported to have occurred in Hungary, near the Austrian frontier. One of them has ended fatally.

A lady named Sampson committed suicide last week by thrusting the handle of a mirror down her throat and causing suffocation.

Hull and Sculcoates Dispensary Committee have passed a motion expressing their deep regret at the death of Alderman Willows, J.P.

The Central London Poor Law Guardians have decided to erect a temporary hospital to hold 400 ophthalmic cases from Hanwell Schools.

Hospital Sunday at Eastbourne resulted in a collection of £425. Not very grand, considering the number of visitors to that town just now.

During 1888 the Warwick Cottage Hospital has received 38 in-patients, and the dispensary attached to it has treated 6,800 out-patients, and made up 20,000 prescriptions.

King's College Hospital nurses scarlet fever, measles, etc., and is the only general hospital which takes infectious cases. All the fevers are nursed in the same ward.

There is news from Montrond, in France, of a plague of butterflies. This week the inhabitants have had to keep their windows and doors shut against an influx of the insects.

The Twickenham friendly and other societies' hospital demonstrations, in aid of St. John's Hospital, Twickenham, and Richmond Hospital, will be held on Saturday, September 7.

A marriage will shortly take place between Deputy Surgeon-General George Alder Watson, Bengal Army (retired), and Beatrice, daughter of the Rev. Canon Holden, D.D., rector of South Luffenham, Rutland.

The Dowager-Empress Augusta visited the Augusta Hospital on the 17th ult., stayed a short time in the Royal Palace Unter den Linden, gave several audiences, and returned to Schloss Babelsberg the same day.

Baron Victor Pereira, a wealthy Austrian nobleman, a descendant of the celebrated Vienna banker, and a member of the Upper Austrian Diet, has recently shown signs of insanity, and is now an inmate of a Vienna lunatic asylum.

The Canadian Government has requested a member of its veterinary staff, Dr. Johnson, who happens to be in Paris, to make some enquiries into the discoveries of M. Pasteur, in connection with innoculation as a preventative of anthrax and pleuro-pneumonia.

At Homerton, on August 29th, Dr. G. C. Miller, the chairman of the Hackney Board of Guardians, laid the foundation-stone of a new block of buildings to be erected at a cost of £20,000 for the purpose of giving increased workhouse accommodation to Hackney.

Droitwich is well filled with visitors, many of whom are taking the brine baths for which this health resort is famous. Staying there at present are Mr. Justice Butt, Dr. Russell, Sir Rutherford and Lady Alcock, Sir Henry Edwards, Major-General Montmorency, and General Ely.

A Roman Catholic priest of Chasetown has accused Mr. Gettings, the medical officer of Lichfield Union, of permitting his unqualified assistant to attend patients, and of signing unsatisfactory certificates of death. Mr. Gettings says the charges arise from personal malice and are unfounded.

